# The influence of caregiver preparedness, perceived social support and general self-efficacy on self-contribution among stroke caregivers: a cross-sectional study

**DOI:** 10.3389/fpubh.2025.1679532

**Published:** 2025-10-16

**Authors:** Hui Teng, Zhou Tan, Mengni Zhen, Qingxia Shu

**Affiliations:** ^1^School of Medicine, Jishou University, Jishou, China; ^2^Department of Neurology and Cerebrovascular Intervention, Hunan University of Medicine General Hospital, Huaihua, China

**Keywords:** stroke, caregiver preparedness, general self-efficacy, perceived social support, self-contribution

## Abstract

**Background:**

Caregivers’ self-care contributions are crucial for enhancing stroke patients’ self-care capabilities, and patients receiving family support often demonstrate higher treatment adherence. Although it is widely recognized that caregivers’ preparedness to provide care positively influences self-care contributions, the underlying mechanisms of this association remain unclear. This study aims to explore the mechanisms through which caregiver preparedness, perceived social support, and general self-efficacy influence caregiver readiness and self-care contribution among stroke caregivers in the contemporary Chinese context.

**Aim:**

Based on the Individual and Family Self-Management Theory (IFSMT) model, this study examined the effects of caregivers’ caregiving preparedness, perceived social support, and general self-efficacy on their self-care contribution among stroke caregivers.

**Design:**

A cross-sectional study.

**Methods:**

Using convenience sampling, 277 stroke caregivers from four tertiary Grade A hospitals in Xiangxi, Hunan Province, China were surveyed with paper-based questionnaires during outpatient follow-up visits in July 2024. Data were analyzed using SPSS/AMOS for descriptive statistics, Pearson correlation, multiple regression, and structural equation modeling with bootstrapping to test hypothesized relationships and mediation effects.

**Results:**

The self-care contribution was at a moderate level, showing significant positive correlations with caregiving preparedness, perceived social support, and general self-efficacy. Both perceived social support and general self-efficacy demonstrated partial mediating effects between caregiving preparedness and self-care contribution.

**Conclusion:**

Improving caregivers’ self-care contribution requires focused attention on enhancing caregiving preparedness, perceived social support, and general self-efficacy. The study highlights the bridging role of perceived social support and general self-efficacy between preparedness and contribution. Clinical interventions should: ① strengthen preparedness through standardized training, ② build social support networks for psychological empowerment, and ③ employ motivational interviewing to boost self-efficacy, thereby establishing a virtuous cycle of “knowledge-resources-confidence”.

## 1 Introduction

Stroke is the leading cause of disability and death among adults in China, with approximately 2 million new cases occurring annually. The incidence is growing at a rate of 8.3%, significantly higher than the global average (1, 2). The China Stroke Prevention and Control Report 2020 indicates that China currently has 17.04 million stroke patients, making it the country with the heaviest global burden of stroke. Among these, individuals aged 60 and above account for 50.81%, approximately 8.65 million people (3). With the accelerating pace of societal aging, it is projected that by 2030, China will have 300 million older adult(s) suffering from cerebrovascular disease (4). Most stroke patients experience significant physiological decline due to the dual impact of neurological deficits and altered psychological states, making it difficult for them to independently perform basic activities of daily living. This severely compromises their health and quality of life, necessitating long-term external support in physical, psychological, and social adaptation. Caregiver self-care contribution refers to the supportive or substitute role caregivers play when patients engage in self-care activities (5). Research indicates that caregiver involvement significantly impacts patients’ self-care processes. The level of self-care contributions made by caregivers can independently influence patients’ self-care behaviors, improve patient outcomes, and reduce the risk of clinical events. This makes caregiver involvement a key factor in achieving favorable health outcomes (6). The self-care contributions of caregivers may be related to their caregiving preparedness (7), general self-efficacy (8), and perceived social support (9).

Caregiver preparedness is considered a key factor influencing caregivers’ self-care contributions. It refers to caregivers’ perceived level of knowledge, skills, and psychological preparedness required to assume caregiving roles and address caregiving challenges (10). This state of readiness can alleviate feelings of caregiving burden while enhancing caregivers’ confidence and capacity to fulfill their caregiving responsibilities. Caregivers for stroke patients are prone to feelings of helplessness and stress due to a lack of caregiving experience, multiple pressures, and social role conflicts. This can diminish their willingness to provide consistent, high-quality care. According to the caregiver stress process model, adequate preparation can effectively buffer the negative effects of caregiving stress. It enhances effective interactions between caregivers, patients, the healthcare system, and the family environment, while improving problem-solving and emotional regulation abilities. This, in turn, promotes more proactive and effective caregiving behaviors.

Perceived social support refers to the various forms of social support that individuals subjectively experience (11), reflecting their satisfaction with their supportive environment. This satisfaction enables individuals to respond to external support by adopting corresponding behaviors, playing a crucial role in facilitating patients’ self-care practices. Research indicates that robust social support can provide respite for caregivers, mitigate the social isolation resulting from caregiving responsibilities, and thereby enhance caregivers’ willingness to engage in patients’ self-care activities.

General self-efficacy refers to an individual’s belief in their ability to effectively organize and execute caregiving tasks and cope with various caregiving challenges. This belief can reduce anxiety when facing difficulties and strengthen caregivers’ determination and perseverance to continue caregiving behaviors. Caregivers of stroke patients are prone to self-doubt and withdrawal due to the complex and unpredictable nature of the condition, the high demands on caregiving skills, and uncertain outcomes, leading to a decline in their motivation to engage in proactive caregiving actions. Research indicates that family caregivers of heart failure patients and Alzheimer’s disease patients with higher levels of self-efficacy adapt more readily to their caregiving roles. They also demonstrate greater willingness to engage in caregiving activities when facing challenges and provide higher-quality care support to patients (8, 12).

The Individual and Family Self-management Theory (IFSMT), proposed by American scholar Ryan snd Sawin (13), focuses on the practices of patients and families in managing their health and illness. It maintains a patient-centered approach while considering the roles of family, friends, and the community. According to IFSMT’s definition, self-management behavior is conceptualized as a multidimensional dynamic system composed of three core elements: context, process, and outcomes. Context is defined as factors that enhance or hinder individual and family participation in self-management, including specific contextual factors (such as disease prevention, treatment, and rehabilitation status), material and social factors (such as healthcare resources and surrounding environment), and individual and family factors (such as personal cognitive state, perspectives, and caregiving capacity). Physical and social environment factors (healthcare resources, surrounding environment). The concept of process encompasses three interacting elements: cognitive and belief systems, self-regulation skills, and social facilitation. Outcomes are categorized into short-term and long-term outcomes: Short-term outcomes primarily manifest in behavioral pattern changes, symptom control levels, medication adherence, and healthcare expenditures; whereas long-term outcomes reflect patients’ physical health status, quality of life scores, and healthcare costs. IFSMT posits that multiple environmental factors not only directly influence the extent to which individuals and their families engage in self-management activities, but also exert indirect effects on both short-term and long-term health outcomes through this process. These contextual factors constitute important external variables that shape the effectiveness of health management, playing a crucial mediating role within the theoretical framework.

Based on the framework of IFSMT, stroke caregivers’ caregiving preparedness, perceived social support, general self-efficacy, and self-care contribution align well with the theoretical constructs, as elaborated below: 1. Caregiving Preparedness corresponds to Personal and Family Factors: The adequacy of preparedness directly impacts the quality and level of care provided, thereby influencing patients’ disease prognosis and quality of life (14). This aligns perfectly with the personal and family factors in IFSMT. 2. General Self-Efficacy corresponds to Belief Systems: As a core element of human behavioral motivation, self-efficacy significantly determines the intensity of individual motivation. Research demonstrates that individuals with higher self-efficacy levels tend to achieve more desirable behavioral outcomes (15), which resonates with the belief systems component in IFSMT. 3. Perceived Social Support corresponds to Social Facilitation: Adequate social support exerts positive influences on individuals, enabling them to better cope with challenges (16). This matches perfectly with the social facilitation dimension in IFSMT. 4. Self-Care Contribution corresponds to Individual and Family Self-Management Behaviors: Enhancing self-care contribution represents a crucial component of caregivers’ participation in patients’ self-management, aiming to improve patients’ self-management capacity and alleviate disease symptoms. This directly reflects the core concept of self-management behaviors in IFSMT.

**Figure 1:**
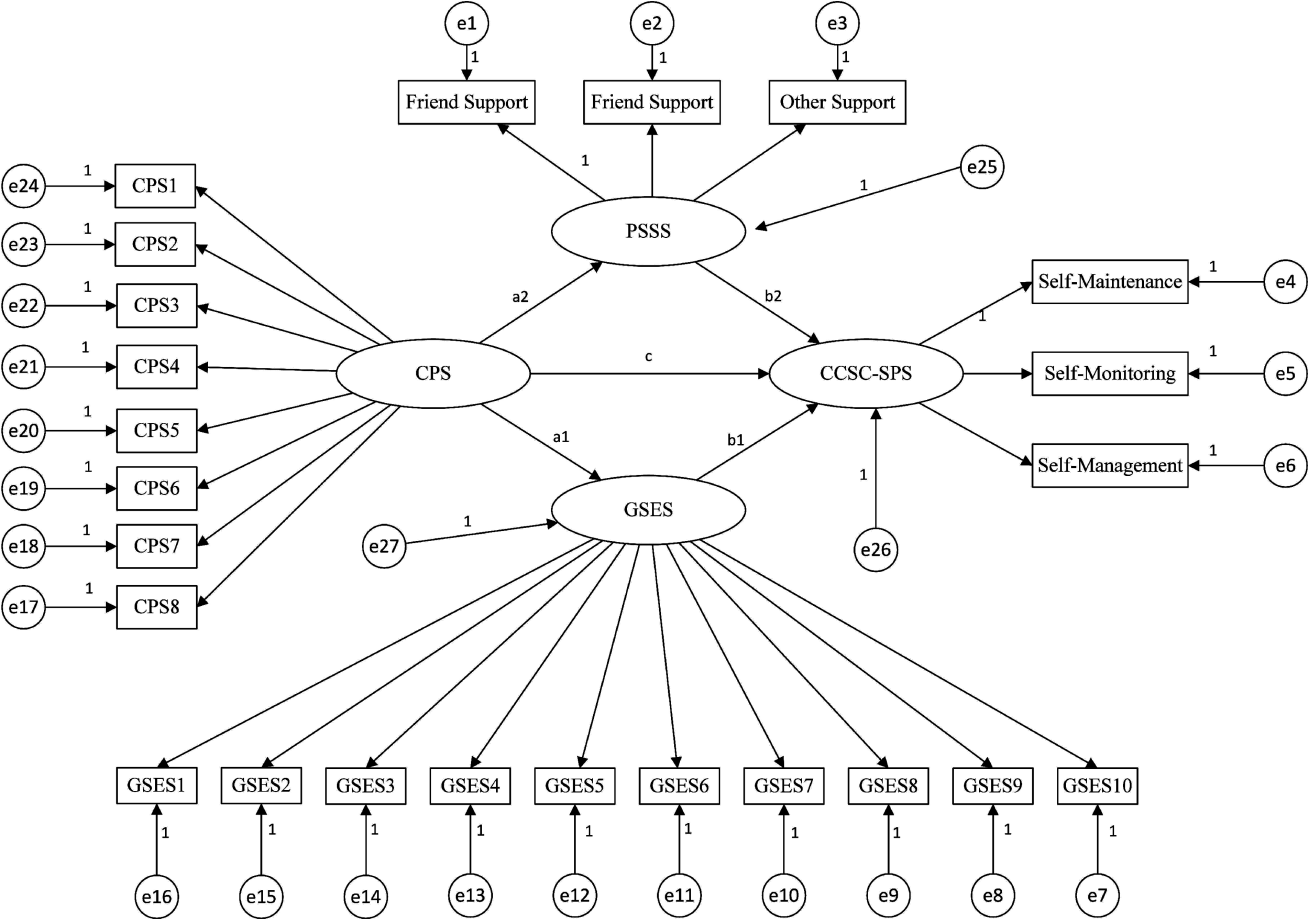
Theoretical model and hypotheses. CPS: Perceived Social Support, GSES: General Self-Efficacy, PSSS: Perceived Social Support, CCSC-SPS: Caregiver Contribution to Self-Care of Stroke Patients.

Based on the theoretical framework of the Individual and Family Self-management Theory model (Figure 1), we propose the following hypotheses:

*H1:* Caregiving preparedness has a direct positive effect on self-care contribution.

*H2:* Caregiving preparedness indirectly affects self-care contribution through general self-efficacy (mediation pathway).

*H3:* Caregiving preparedness indirectly affects self-care contribution through perceived social support (mediation pathway).

*H4:* Perceived social support has a direct positive effect on self-care contribution.

*H5:* General self-efficacy has a direct positive effect on self-care contribution.

## 2 Materials and methods

### 2.1 Study design

This study was a hospital-based, multicenter, cross-sectional survey using convenience sampling and followed the Strengthening the Reporting of Observational Studies in Epidemiology (STROBE) guidelines for reporting (17).

The sample size was determined using Kendall’s rough estimation method (18): sample size (*N*) = Number of research variables (*n*) × (5−10). The selected survey instruments included: General Information Questionnaire (18 items), Caregiver Contribution to Self-care of Stroke Patients Scale (7 dimensions), Caregiver Preparedness Scale (1 dimension), Perceived Social Support Scale (3 dimensions), General Self-efficacy Scale (1 dimension) (Table 1). With a total of 30 independent variables and considering a 20% buffer for invalid questionnaires, the estimated sample size range was 180–360 participants.

**Table 1:** Operational definition of variables.

Variables	Operational definition
caregiving preparedness	In this study, the term refers to the level of caregiving preparedness among caregivers of stroke patients, measured using the Caregiver preparedness Scale.
Perceived Social Support	In this study, the perceived level of social support among caregivers of stroke patients was measured using the Perceived Social Support Scale.
General Self-Efficacy	In this study, it refers to the general self-efficacy level of caregivers for stroke patients, measured using the General Self-Efficacy Scale.
Caregiver Contribution to Self-Care of Stroke Patients	In this study, it refers to the level of self-care contribution by caregivers of stroke patients, measured using the Caregiver Contribution to Self-Care of Stroke Patients Scale.

### 2.2 Participants

This study is a multicenter cross-sectional investigation aimed at examining the current state of self-care contributions among caregivers of stroke patients and analyzing the path relationships between these contributions and caregiving preparedness, perceived social support, and general self-efficacy. Researchers recruited 277 caregivers of stroke patients from outpatient departments of three geographically distinct Grade A tertiary general hospitals in Xiangxi, Hunan Province, China, between October 2023 and July 2024. The specific sample origins were: Huaihua City, Hunan (137 cases), Jishou City, Hunan (70 cases), and Zhangjiajie City, Hunan (70 cases). The aforementioned research areas are all located within the Wuling Mountain region, forming the core zone of the contiguous special poverty-stricken area at the junction of Hunan, Hubei, Chongqing, and Guizhou provinces. These three regions exhibit pronounced urban–rural dual structures, high proportions of rural populations, and concentrated ethnic minority communities. Collectively serving over ten million residents, they possess exceptional typicality and representativeness for the development of ethnic minority mountainous areas in central and western China, effectively reflecting the common characteristics of similar regions. Inclusion criteria for patients: 1. According to Chinese Guidelines for Diagnosis and Treatment of Acute Ischemic Stroke (2023 Edition) and Chinese Guidelines for Diagnosis and Treatment of Cerebral Hemorrhage (2019 Edition) Diagnosed with stroke by clinicians (19, 20); 2. Aged ≥18 years; 3. In the recovery phase of illness (defined in this study as stroke patients within 1 to 6 months after hospital discharge); 4. Accompanied by 1–2 caregivers for outpatient follow-up visits.; 5. Normal communication, expression, reading and writing abilities. Inclusion criteria for caregivers: 1. Aged ≥18 years; 2. Continuous care duration >1 month; 3. Normal cognitive and language functions Willing to provide informed consent and participate voluntarily. Exclusion Criteria for Patients: 1. Comorbid with critical illnesses or severe trauma; 2. Hemorrhagic stroke caused by trauma; 3. Transient ischemic attack (TIA) patients. Exclusion Criteria for Caregivers: 1. Receiving remuneration for caregiving; 2. Suffering from severe health impairment; 3. Concurrent participation in other studies.

### 2.3 Data collection

The interviewer explains the purpose and significance of the survey to the respondent using standardized instructions. After obtaining consent, the interviewer explains how to complete the questionnaire and any important considerations. Once the questionnaire is completed, it is verified on the spot to ensure validity. A total of 280 questionnaires were distributed for this survey, achieving a 100% return rate. After rigorous screening, three questionnaires with incomplete responses or logical errors were excluded. This resulted in 277 valid questionnaires, yielding an effective return rate of 98.9%, which met the required sample size for the study.

### 2.4 Measures

#### 2.4.1 Participants’ general characteristics

Caregiver demographic characteristics: age, gender, occupation, marital status, education level, relationship to patient, cohabitation status with patient, daily caregiving duration, monthly household income. Patient demographic characteristics: gender, age, marital status, education level, residence location, healthcare payment method, number of comorbid chronic conditions, hospitalization frequency: first admission, stroke type: ischemic stroke.

#### 2.4.2 Caregiver contribution to self-care of stroke patients scale (CCSC-SPS)

The CCSC-SPS was developed by Chinese scholar Wang et al. (21) to specifically assess caregivers’ contributions to stroke patients’ self-care. The scale consists of three subscales (contribution to self-care maintenance, contribution to self-care management contribution to self-care monitoring), seven dimensions, and a total of 23 items. It uses a 5-point Likert scale, with a total score ranging from 23 to 115 points. Higher scores indicate a higher level of contribution by caregivers to patients. In the present study, the scale showed excellent internal consistency (Cronbach’s α = 0.956).

#### 2.4.3 General self-efficacy scale (GSES)

The GSES was developed by Professor Schwarzer et al. in Germany. Wang et al. (22) adapted, translated, and revised it for Chinese use. Utilizing a 4-point Likert scale (total score range: 10–40), higher scores indicate stronger self-confidence and perceived competence in performing caregiving tasks. In the present study, the scale showed excellent internal consistency (Cronbach’s α = 0.951).

#### 2.4.4 Perceived social support scale (PSSS)

The PSSS was originally developed by Zimet et al., with the Chinese version translated and culturally adapted by scholar Qin et al. (23). The 12-item instrument assesses three dimensions: friend support, family support, and other support, using a 7-point Likert scale. Higher total scores indicate stronger perceived support availability and adequacy within one’s social network. In the current study, the scale demonstrated excellent reliability (Cronbach’s α = 0.954).

#### 2.4.5 Caregiver preparedness scale (CPS)

The CPS was originally developed by Archbold et al. (10), with its Chinese version subsequently translated and validated by Chinese scholar Liu et al. (24). The 8-item instrument uses a 5-point Likert scale (total score range: 0–32), where higher scores indicate greater psychological and competency preparedness for caregiving responsibilities. In this study, the scale demonstrated excellent internal consistency (Cronbach’s*α* = 0.957).

### 2.5 Statistical analysis

The study employed SPSS 23.0 and AMOS 24.0 for statistical analysis with a two-tailed significance level of α = 0.05: (1) Descriptive statistics were performed on general characteristics—normally distributed continuous variables were presented as mean ± standard deviation, non-normally distributed continuous variables as median (interquartile range), and categorical variables as frequencies (percentages); (2) ANOVA and t-tests analyzed differences in caregivers’ self-care contribution across demographic variables (statistical significance at *p* < 0.05); (3) Pearson correlation analyzed relationships between self-care contribution and caregiving preparedness, perceived social support, and general self-efficacy; (4) multiple linear regression modeled self-care contribution (dependent variable) against total scores of preparedness, social support, self-efficacy, and statistically significant univariate factors (independent variables); (5) AMOS 24.0 tested the theoretical model, examining pathways and effect sizes among preparedness, social support, self-efficacy, and self-care contribution.

### 2.6 Ethical considerations

The research protocol must be submitted to the Medical Ethics Committee of Jishou University for review and evaluation. Subsequent data collection may only proceed after obtaining formal approval documents (approval no.: JSDX-2024-0052). During data collection, researchers must provide stroke caregivers with detailed explanations of the study protocol, including research objectives, implementation procedures, data confidentiality measures and other key information. Surveys may only be conducted after obtaining their informed consent. Simultaneously, caregivers must be explicitly informed of their right to suspend participation or withdraw from the study at any time, thereby fully protecting their autonomous decision-making rights.

## 3 Results

### 3.1 Characteristics of the sample

#### 3.1.1 Demographic characteristics of stroke caregivers

The average age of the 277 stroke caregivers was (53.06 ± 12.77) years. The largest age group was 41 ~ 59 years old, comprising 123 individuals (44.2%). Gender: Females predominated, with 180 individuals (65.0%). Marital status: Married individuals were the majority, with 256 individuals (92.4%). Occupation: Farmers constituted the largest occupational group among primary caregivers, with 112 individuals (40.4%). Monthly household income: The largest income bracket was >5,000 yuan, with 124 individuals (44.8%). See Table 2 for details.

**Table 2:** Demographic characteristics of stroke caregivers (*n* = 277).

Variables	Groups	*N*(%)	CCSC-SPS (x̅ ± s)	*F/t*	*p*-value
Age (years)	≤40	53 (19.1)	69.02 ± 20.98	4.275	0.006
	41 ~ 49	58 (20.9)	74.48 ± 23.04		
	50 ~ 59	65 (23.5)	71.54 ± 16.32		
	≥60	101 (36.5)	63.85 ± 18.07		
Gender	Male	97 (35.0)	65.15 ± 21.50	−2.214	0.028
	Female	180 (65.0)	70.87 ± 18.51		
Marital Status	Single	11 (4.0)	69.09 ± 21.29	0.780	0.506
	Married	256 (92.4)	69.06 ± 19.56		
	Divorced	6 (2.2)	70.83 ± 27.75		
	Widowed	4 (1.4)	54.00 ± 17.22		
Occupation	Farmer	112 (40.4)	65.49 ± 19.43	1.874	0.134
	Self-employed	42 (15.2)	71.45 ± 16.78		
	Government employee	42 (15.2)	69.02 ± 20.98		
	Other (retired, etc.)	81 (29.2)	70.75 ± 21.02		
Monthly household income	<2,000	58 (20.9)	64.45 ± 23.45	3.357	0.036
	2,000 ~ 5,000	95 (34.3)	67.43 ± 18.48		
	>5,000	124 (44.8)	72.04 ± 18.42		
Education level	Primary school or below	76 (27.4)	62.75 ± 20.24	4.485	0.004
	Middle school	82 (29.6)	68.94 ± 20.29		
	High school	73 (26.4)	71.21 ± 17.67		
	Associate degree or higher	46 (16.6)	75.15 ± 18.95		
Daily caregiving hours	4 ~ 8 h	49 (17.7)	69.94 ± 20.09	0.283	0.753
	8 ~ 12 h	86 (31.0)	69.70 ± 19.13		
	>12 h	142 (51.3)	68.00 ± 20.11		
Relationship to patient	Spouse	151 (54.5)	71.30 ± 18.44	3.274	0.039
	Child	99 (35.7)	67.04 ± 20.40		
	Other (parent, friend, etc.)	27 (9.7)	61.96 ± 22.73		
Cohabitation with patient	Yes	225 (81.2)	69.12 ± 19.84	0.437	0.662
	No	52 (18.8)	67.79 ± 19.55		

#### 3.1.2 Demographic characteristics of stroke patients

The mean age of 277 stroke patients was (64.13 ± 12.46) years. The largest age group was ≥60 years, comprising 186 patients (67.1%). Gender distribution showed a male predominance with 175 patients (63.2%). Marital status revealed a majority of married individuals, totaling 214 patients (77.3%). Stroke type: Ischemic stroke was predominant, affecting 210 patients (75.8%). See Table 3 for details.

**Table 3:** Demographic characteristics of stroke patients (*n* = 277).

Variables	Groups	*N* (%)	CCSC-SPS (x̅ ± s)	*F/t*	*p*-value
Age (years)	<40	17 (6.1)	57.00 ± 20.83	5.332	0.001
	41 ~ 49	23 (8.3)	59.00 ± 23.43		
	50 ~ 59	51 (18.4)	67.76 ± 19.74		
	≥60	186 (67.1)	71.48 ± 18.49		
Gender	Male	175 (63.2)	67.94 ± 20.43	−1.056	0.292
	Female	102 (36.8)	70.47 ± 18.54		
Marital status	Single	15 (5.4)	74.93 ± 20.09	1.049	0.371
	Married	214 (77.3)	69.04 ± 20.00		
	Divorced	9 (3.2)	71.67 ± 14.24		
	Widowed	39 (14.1)	64.95 ± 19.22		
Education level	Primary school or below	116 (41.9)	68.38 ± 19.88	0.928	0.427
	Middle school	68 (24.5)	71.38 ± 17.82		
	Technical secondary or high school	58 (20.9)	69.43 ± 20.72		
	Junior college or above	35 (12.6)	64.69 ± 21.34		
Stroke type	Hemorrhagic stroke	67 (24.2)	68.39 ± 20.76	−0.229	0.819
	Ischemic stroke	210 (75.8)	69.02 ± 19.48		
Number of hospitalizations	First admission	173 (62.5)	65.92 ± 19.40	9.546	<0.001
	2 admissions	87 (31.4)	71.40 ± 18.22		
	≥3 admissions	17 (6.1)	85.94 ± 21.57		
Number of chronic comorbidities	≤1 comorbidity	117 (42.2)	63.50 ± 20.00	15.610	<0.001
	2 comorbidities	132 (47.7)	70.18 ± 18.03		
	≥3 comorbidities	28 (10.1)	85.14 ± 16.76		
Payment method	Out-of-pocket	31 (11.2)	69.23 ± 19.86	0.458	0.747
	Urban–rural resident medical insurance	104 (37.5)	69.02 ± 20.74		
	Employee medical insurance	63 (22.7)	70.27 ± 20.30		
	New rural cooperative medical scheme	77 (27.8)	67.03 ± 18.24		
	Other (commercial insurance, etc.)	2 (0.7)	82.50 ± 4.95		
Residence	Rural	110 (39.7)	67.56 ± 18.86	−0.893	0.373
	Urban	167 (60.3)	69.73 ± 20.34		

### 3.2 Common deviation test

Harman’s single-factor test method was used to test the input common deviation of variables in each scale (32). The results showed that there were 21 factors with characteristic roots greater than 1 extracted by the factors. The explanation rate of the first common factor was 37.67%, which was less than the critical value of 40.00%, indicating that the common deviation of this study was not significant and the research results are credible.

### 3.3 Correlation between caregiving preparedness, perceived social support, general self-efficacy scores, and self-care contributions in stroke caregivers

The scores of caregiving preparedness, perceived social support, and general self-efficacy and self-care contributions Scores were (14.32 ± 8.51), (49.39 ± 18.35), (20.52 ± 6.84), and (68.87 ± 19.76) respectively. The mean and difference of caregiving preparedness, perceived social support, and general self-efficacy and self-care contributions, and the Pearson correlation test results between the variables are shown in Table 4.

**Table 4:** Correlation between caregiving preparedness, perceived social support, general self-efficacy scores, and self-care contributions in stroke caregivers (*n* = 277).

Variables	Score (x̅ ± s)	1	2	3	4
Caregiving preparedness	14.32 ± 8.51	1	-	-	-
Perceived social support	49.39 ± 18.35	0.394**	1	-	-
General self-efficacy	20.52 ± 6.84	0.393**	0.408**	1	-
Self-care contribution	68.87 ± 19.76	0.412**	0.475**	0.412**	1

**p < 0.01; all tests were two-tailed.

Significant positive correlations were observed between stroke caregivers’ caregiving preparedness, perceived social support, general self-efficacy and their self-care contribution (r = 0.412, 0.475, 0.412 respectively, *p* < 0.01). See Table 5 for details.

**Table 5:** Effect analysis of influencing factors on self-care contribution among stroke caregivers.

Variables	Effect value (*β*)	Share of total effect (%)	95%CI	*p*-value
Total effect	0.448	100%	0.340 ~ 0.548	<0.001
Total indirect effect	0.253	56.47	0.010 ~ 0.275	<0.001
Direct effect (CPS → CCSC-SPS)	0.195	43.53	0.057 ~ 0.326	0.005
CPS → GSES→CCSC-SPS	0.048	10.71	0.015 ~ 0.101	<0.001
CPS → PSSS→CCSC-SPS	0.178	39.73	0.103 ~ 0.275	<0.001
CPS → PSSS→GSES→CCSC-SPS	0.027	6.03	0.010 ~ 0.055	0.005

### 3.4 Structural model

This study employed the bias-corrected Bootstrap method (Bootstrap samples = 5,000) for mediation analysis. All continuous variables were standardized and controlled for covariates significant in the univariate analysis.

Based on the IFSMT, a structural equation model was constructed to examine the factors influencing self-care contribution among caregivers of stroke patients. This model treated caregiving preparedness as an exogenous latent variable, perceived social support and general self-efficacy as mediating latent variables, and self-care contribution as the outcome variable. The model demonstrated an acceptable fit: χ^2^/df = 2.051 < 3, RMSEA = 0.062, CFI = 0.953, TLI = 0.947, and IFI = 0.953.

### 3.5 Model results

**Figure 2:**
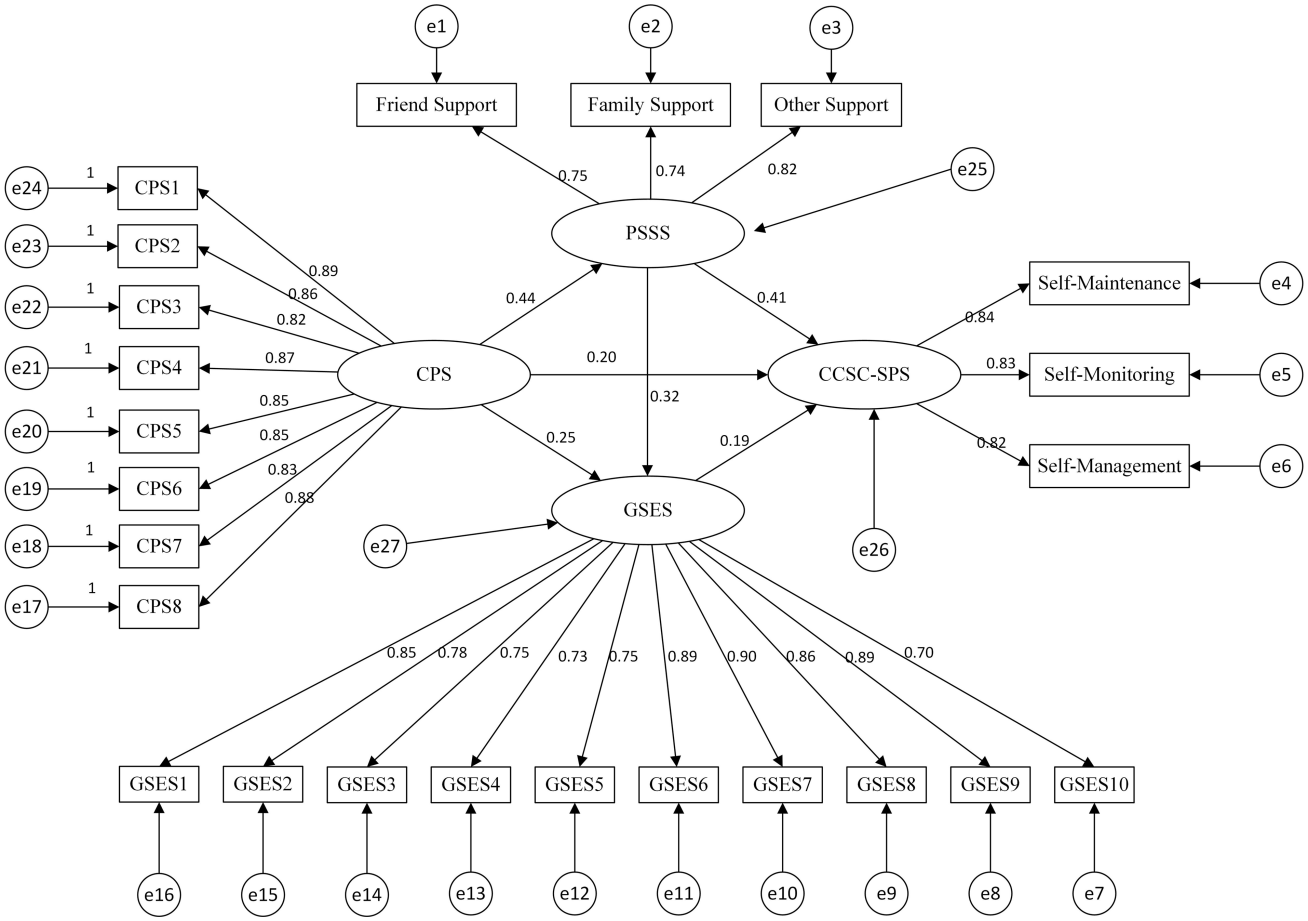
Final model and standardised model paths. Solid lines indicate significant paths; path coefficients are standardised coefficients. The model controls age, number of hospitalizations, gender, marital status, education level, monthly household income, number of chronic comorbidities and relationship to patient.

Caregivers’ caregiving preparedness to provide care has a direct positive effect on their self-care contribution (*β* = 0.195) and exerts an indirect effect through three pathways, accounting for 56.47% of the total effect (Figure 2).

## 4 Discussion

Caregiving preparedness, perceived social support, and general self-efficacy positively contribute to enhancing self-care contributions among stroke caregivers. Perceived social support and general self-efficacy partially mediate the relationship between caregiving preparedness and self-care contributions. From the perspective of the IFSMT, these findings suggest that the aforementioned factors may enhance caregivers’ self-care contributions by influencing behavior changes related to their caregiving preparedness. This discovery deepens our understanding of the underlying mechanisms and pathway relationships among the various variables.

### 4.1 Self-care contributions among caregivers of stroke patients are at a moderately low level

Research findings indicate that caregivers of stroke patients achieved a total self-care contribution score of (68.87 ± 19.76), which falls below the midpoint of 75 on the scale, indicating a moderately low level. This finding is lower than the results of Wang Bingbing’s survey on caregivers of hospitalized stroke patients (25). Possible reasons for this discrepancy include: (1) Overall low educational attainment. In this study, 57% of caregivers had an educational level of junior high school or below. It is challenging for them to comprehensively understand and apply knowledge and skills related to home-based stroke care. Caregivers often feel overwhelmed and profoundly helpless due to this lack of knowledge and skills, hindering their effective participation in the patient’s self-care practices. (2) Differences in study populations: Wang Bingbing’s research exclusively included spouses as subjects, whereas this study incorporated children and friends of patients. Compared to spouses, these individuals often overlook the importance of self-care maintenance contributions to promoting patient recovery due to their own family responsibilities, work commitments, leisure activities, and other factors. (3) The prevalence of comorbid chronic conditions differs. In Wang Bingbing et al.’s study, only 24.5% of patients had chronic illnesses, whereas this study found a significantly higher prevalence of 57.8%. Research indicates that patients may spontaneously engage in better self-care maintenance behaviors due to their chronic disease status, while the interdependence between patients and caregivers may lead to lower caregiver contributions (26). In summary, healthcare providers should prioritize attention to this category of caregivers, promptly assess their level of self-care contribution, and develop comprehensive, problem-solving-oriented psychological interventions. This includes providing disease-specific caregiving information and skills training to further enhance their self-care contribution, thereby enabling them to better care for patients.

### 4.2 Caregiving preparedness can directly influence self-care contributions

This study demonstrates that caregiving preparedness directly influences self-care contributions, consistent with the findings of Vellone’s research (7). Family caregivers must prepare for caregiving tasks, including providing daily living assistance, psychological support, and managing emergencies, to meet patients’ physical and psychological needs. When these needs are fulfilled, patients’ treatment adherence, confidence in recovery, and self-care management behaviors significantly improve. This indirectly reduces caregivers’ nursing burden and enhances their confidence in participating in patients’ self-care. Therefore, healthcare providers should proactively introduce diverse support channels to family caregivers, including peer support platforms, self-management education courses, specialized health lectures, and systematic post-discharge follow-up services. This approach enhances caregivers’ preparedness, strengthens their confidence in providing care, and ultimately promotes the patient’s recovery process.

### 4.3 Caregiving preparedness can indirectly influence self-care contribution through perceived social support and general self-efficacy

#### 4.3.1 Caregiving preparedness may indirectly influence self-care contributions through perceived social support

Caregiving preparedness indirectly influences self-care contributions through perceived social support, with the mediating effect accounting for 39.73% of the total effect. Perceived social support refers to the degree to which an individual feels respected, understood, and supported within their social environment, serving as a significant factor influencing caregivers’ self-care contributions (27). Caregivers with lower preparedness levels who lack social support from friends, family, and relatives are prone to developing negative emotions, which hinders their ability to increase their self-care contributions. Therefore, healthcare providers should enhance caregivers’ preparedness and meet their perceived social support needs based on the interrelationship between caregiving preparedness, perceived social support, and self-care contributions. This approach aims to elevate self-care contributions through the mediating effect of perceived social support, thereby improving patients’ treatment adherence and self-care behaviors.

#### 4.3.2 Caregiving preparedness may indirectly influence self-care contributions through general self-efficacy

Caring preparedness can indirectly influence self-care contributions through general self-efficacy, with the mediating effect accounting for 10.73% of the total effect. Caregivers with high self-efficacy typically exhibit a positive and optimistic mindset. During the caregiving process, they tend to possess greater self-confidence and self-esteem, maintaining a positive outlook on their own behaviors and capabilities. This helps them regulate their emotions, enabling them to better engage in the patient’s self-care activities. Healthcare professionals can enhance caregivers’ understanding of stroke and their caregiving skills through health education lectures and training workshops, thereby boosting their confidence in caring for patients.

#### 4.3.3 Perceived social support and general self-efficacy exert a chained mediating effect between caregiving preparedness and self-care contributions

Unlike the variable relationships identified in the IFSMT model, this study introduces an additional pathway: “perceived social support → general self-efficacy.” This indicates that caregivers’ caregiving preparedness to provide care can also influence self-care contributions through the chained mediating effects of perceived social support and general self-efficacy, with the mediating effect accounting for 6.03% of the total effect. High levels of social support can foster a sense of identity and belonging among caregivers, enhance their positive emotions, and boost their self-confidence, thereby encouraging them to actively participate in the patient’s self-management of their condition. Clinical healthcare teams should prioritize strengthening caregivers’ social support systems. By enhancing caregivers’ sense of involvement and responsibility, their proactive engagement can be stimulated. This approach increases the positive impact of caregiving preparedness on self-care contributions, thereby further elevating their level of self-care engagement.

## 5 Conclusion

This study systematically analyzed the key factors influencing stroke caregivers’ self-care contributions based on the Individual and Family Self-Management Theory (IFSMT) framework. The results indicate that caregiver preparedness, perceived social support, and general self-efficacy are the three core factors enhancing self-care contribution levels, with significant synergistic effects among them: the higher the caregiver preparedness, the stronger the perceived social support, and the greater the confidence in participating in the patient’s self-care, the more prominent the caregiver’s role in the patient’s self-management. Based on these findings, we recommend developing a multidimensional intervention system guided by the IFSMT framework: (1) Enhancing caregivers’ disease management knowledge and skills through standardized training to strengthen their preparedness; (2) Using motivational interviewing and other techniques to improve caregivers’ general self-efficacy; (3) Establishing a social support network involving family members and healthcare professionals. This tripartite intervention strategy can effectively enhance caregivers’ self-care contributions, thereby improving patients’ self-care behaviors.

Overall, employing IFSMT to investigate factors influencing self-care contributions among caregivers of stroke patients holds dual significance: First, this method systematically identifies key variables affecting caregivers’ self-care contributions; second, it clearly elucidates the interactive mechanisms among these variables and their pathways of influence on caregivers’ self-care contributions. Compared to traditional regression analysis methods, this theoretical framework offers a more comprehensive and in-depth research perspective, representing a significant expansion and complement to quantitative analytical approaches.

## 6 Limitations

This study has several limitations. Firstly, the small sample size and reliance on convenience sampling in this study limited the generalizability of the findings. Future research could employ random sampling or stratified sampling methods to ensure the representativeness and comparability of the sample. Additionally, expanding the sample size, increasing the number of participants, and diversifying the study variables would enhance the reliability and universality of the results, thereby providing a better understanding of the attitudes and behaviors of the subjects. Secondly, due to the cross-sectional study design, this research only reflects the current status of self-care contributions among stroke caregivers at a specific point in time and cannot demonstrate longitudinal changes in their self-care contribution levels. Follow-up studies could adopt a longitudinal approach to observe the dynamic patterns of stroke caregivers’ self-care contributions over time and explore the relevant influencing factors. Finally, although this study has made every effort to distinguish these concepts in measurement, we note that ‘caregiving preparedness,’ ‘general self-efficacy’, and ‘self-care contribution’ are closely interrelated in their theoretical implications, which may introduce certain limitations. Future research may employ longitudinal designs to further clarify the causal pathways among these concepts, thereby revealing their underlying mechanisms with greater precision.

## Data Availability

The raw data supporting the conclusions of this article will be made available by the authors, without undue reservation.
